# Relationship between postablation fever and prognosis in initial hepatocellular carcinoma: a 15-year multicenter, retrospective cohort study

**DOI:** 10.1097/JS9.0000000000002066

**Published:** 2024-09-18

**Authors:** Qian Cai, Chuan Pang, Zhen Wang, Jianming Li, Yuqing Dai, Fang-ying Fan, Zhong-qi Wang, Xin Hu, Lijuan Li, Xu-wei Chen, Ran Ji, Qian Mei, Chao Zhang, Ping Liang, Xiaoling Yu, Fang-yi Liu, Zhigang Cheng, Jie Yu

**Affiliations:** a Senior Department of Oncology, Department of Interventional Ultrasound, The Fifth Medical Center of PLA General Hospital, Beijing, People’s Republic of China; b Department of Orthopedics, Fourth Medical Center of Chinese PLA General Hospital and Chinese PLA Medical College, Beijing, People’s Republic of China; c Departments of Frontier Surgery, Graduate School of Medicine, Chiba University, Chiba, Japan; d Department of Breast and Thyroid Surgery, Qingdao Women and Children’s Hospital, Qingdao University, Qingdao, People’s Republic of China; e Department of Bio-Therapeutic, The First Medical Center of Chinese PLA General Hospital, Beijing, People’s Republic of China; f Senior Department of Infectious Diseases, The Fifth Medical Center of PLA General Hospital, Beijing, People’s Republic of China

**Keywords:** early recurrence, hepatocellular carcinoma, lymphocyte count, postablation fever, prognosis

## Abstract

**Background:**

Fever is a common side effect following thermal ablation in patients with hepatocellular carcinoma (HCC), yet its impact on prognosis remains unclear.

**Materials and Methods:**

This retrospective study included initial HCC patients who underwent US-guided percutaneous microwave ablation at 13 hospitals between January 2006 and February 2021. All patients were categorized into afebrile, transient low-grade fever (TLF), and prolonged or high-grade fever (PHF) groups. Primary outcomes included very early recurrence (VER) and early recurrence (ER), secondary outcomes were disease-free survival (DFS) and overall survival (OS). Fever cut-offs for VER/ER were established using restrictive cubic splines and an adjusted Cox model. Survival analyses used the Kaplan–Meier method.

**Results:**

A total of 1458 initial HCC patients (mean age, 59±11[SD]; 1146 men). Compared to afebrile individuals, patients with TLF (temperatures ranging 37.0–38.8°C for 1–2 days), showed independent protective effects against VER (HR, 0.73; 95% CI: 0.57–0.95; *P*=0.02) and ER (HR, 0.66; 95% CI: 0.54–0.81; *P*<0.001), however, PHF showed no differences in VER (HR, 0.99; 95% CI: 0.76–1.30; *P*=0.96) and ER (HR, 0.86; 95% CI: 0.69–1.07; *P*=0.17). With a median follow-up of 47 months (IQR: 26–79), the median DFS for TLF patients was 40 months, superior to afebrile (30 months, *P*=0.019) and PHF patients (33 months, *P*=0.049). The 5-year OS rate for TLF patients was 73.2%, higher than afebrile (69.3%, *P*=0.02) and PHF patients (66.7%, *P*=0.03). No significant difference was found in DFS and OS between afebrile and PHF patients (*P*=0.90 and 0.71). Notably, TLF patients exhibited the highest lymphocyte counts increasing median 7 days after ablation (*P*<0.001 vs. afebrile and *P*=0.01 vs. PHF).

**Conclusion:**

Transient low-grade fever following percutaneous microwave ablation in hepatocellular carcinoma patients demonstrated protection against early recurrence, possibly attributed to the short-term activation of lymphocytes.

## Introduction

Highlights

- Statistical models identified a correlation between fever in initial HCC patients after curative ablation and early recurrence.
- Transient low-grade fever patients exhibited the lowest early recurrence rates, the longest median disease-free survival, and the highest 5-year overall survival.
- Transient low-grade fever patients showed the highest postfever lymphocyte count increase.
- Afebrile patients showed the highest preablation neutrophil-to-lymphocyte ratio (NLR), while the prolonged or high-grade fever patients showed the highest NLR at 1 day and median 7 days postablation.

Hepatocellular carcinoma (HCC), being the most common primary liver cancer, is a major contributor to global cancer-related deaths^1–3^. Surgical resection, ablation, transarterial chemoembolization (TACE), and liver transplantation are the main options for HCC treatment^4^. Among these, image-guided ablation, known for its minimally invasive procedure and comparable survival outcomes to resection, has gained widespread acceptance as the recommended first-line treatment for early-stage HCC worldwide^4–6^. Furthermore, increasing studies have shown a ‘treatment transition’, supporting the move from TACE to ablative therapies for select cases of intermediate-stage HCC, offering a promising curative treatment strategy for these patients^7–9^.

In recent years, advances in equipment and technique have made microwave ablation (MWA) the newest and most effective method in the field of thermal ablation for HCC^10–12^. However, due to the high heterogeneity and suppressive nature of HCC, neither ablation nor surgical resection can effectively prevent its frequent recurrence^13^. The annual recurrence rate for HCC within the Milan criteria after complete ablation is ≥10%, and it reaches 70–80% after 5 years^14^. Differing from resection, thermal ablation not only precisely eradicates tumors in situ but also generates heat stimulation, triggers the release of immunogenic intracellular substrates and cytokines, and further activates innate immunity and acquired responses^15–17^. Postablation fever, the most common side effect following ablation, is predominantly self-limiting and closely associated with cellular damage and inflammatory responses after treatment^18,19^. Mounting evidence suggests that fever, within the range of ΔT~1–4°C above baseline, can enhance multiple innate and adaptive immune defense mechanisms in individuals with cancer, thereby potentially increasing the survival rates^20^. However, the excessive release of chemokines can result in the infiltration of immunosuppressive cells and the formation of new blood vessels within tumors, potentially promoting tumor growth^21^.

The levels of inflammation and immune responses can vary significantly under different fever conditions, and fever itself can result from the complex interaction between inflammation and the immune system in the body^18,22,23^. Investigating which type of postablation fever serves as a protective factor or poses a risk is of clinical significance. To date, and to our knowledge, no large-scale research has addressed this matter.

The aim of this study was to investigate the correlation between postablation temperature variations and the recurrence and survival of HCC patients, while exploring alterations in lymphocyte and neutrophil profiles among groups with different temperature changes.

## Material and methods

### Patients and study design

This retrospective cohort study collected data from 13 tertiary medical centers covering a wide geographical area in China. It included 1978 consecutive diagnosed initial HCC patients who underwent MWA as their first-line treatment between January 2006 and February 2021. This multicenter study protocol was approved by the ethics committee of the primary research unit (No: S2012-020) and adhered to the principles outlined in the 1975 Declaration of Helsinki. It was registered on ClinicalTrials.gov. The findings have been reported following the guidelines set forth in the strengthening the reporting of cohort, cross-sectional, and case–control studies in surgery (STROCSS, Supplemental Digital Content 1, http://links.lww.com/JS9/D449) criteria^24^.

The inclusion criteria for the patient enrollment were as follows: (1) age ≥18 years; (2) initial HCC with a confirmed pathological diagnosis; (3) Barcelona Clinic Liver Cancer (BCLC) stage 0-B with all HCC nodules measuring ≤5 cm in maximum diameter and ≤3 nodules in total (for curative treatment); (4) Child-Pugh class A or B; (5) no other malignancy before ablation; and (6) prothrombin time ratio >50% (prothrombin time with an international normalized ratio <1.7) and platelet count >40×10^9^/l.

We excluded patients with incomplete ablation and incomplete follow-up data, as well as those who experienced fever due to postablation bleeding or secondary infection.

### Microwave ablation procedures

All MWA procedures were performed percutaneously under US guidance (Signature 7.2, Acuson Sequoia 512; Siemens Medical Solutions or LOGIQ E9; GE Medical Systems US and Primary Care Diagnostics). Names of the 13 hospitals and the experience of the 36 operators are described in Table S1 (Supplemental Digital Content 2, http://links.lww.com/JS9/D450) and Appendix S1 (Supplemental Digital Content 2, http://links.lww.com/JS9/D450). Details of the procedure and application of assistive technologies are presented in Appendix S2 (Supplemental Digital Content 2, http://links.lww.com/JS9/D450).

### Data collection and follow-up

During the patient’s hospitalization, body temperature was measured every 4–6 hours. For patients with a fever, more frequent monitoring was conducted, with measurements taken every 1–2 h during the daytime. All temperature readings were documented in a record sheet. Dynamic monitoring of peripheral complete blood count, including absolute lymphocyte count (ALC) and neutrophil count, commenced from postablation day 1 until the day of discharge.

An early postprocedure assessment was conducted within 3 days after MWA, using contrast-enhanced MRI or CT scans to evaluate the completeness of the ablation and identify potential complications such as bleeding or fluid accumulation. Subsequent follow-up evaluations were scheduled at 1, 3, and 6-month intervals until death after MWA, which included enhanced MRI/CT scans and measurements of serum α-fetoprotein (AFP). If local tumor progression (LTP) or intrahepatic distant metastasis (IDM) was observed during subsequent follow-up visits, an additional MWA was performed within a month after new lesions were detected, if possible. Chest radiographs and other appropriate examinations were performed for the detection of extrahepatic metastasis (EM). The definitions of LTP, IDM, and EM are based on the standardization by the International Working Group on Image-Guided Tumor Ablation^25^. LTP is identified by the emergence of tumor foci at the edge of the ablation zone after adequate ablation has been confirmed by at least one contrast-enhanced image, showing no viable tissue in the target tumor and its surrounding margin. IDM refers to a new lesion within the liver that does not contact the original ablation zone. EM is defined as tumor seeding or the development of new lesions outside the liver.

### Study outcomes

Tumor progression comprised LTP, IDM, or EM based on new lesion location. The primary outcomes were very early recurrence (VER), defined as tumor progression occurring within 1 year after MWA, and early recurrence (ER), defined as tumor progression within 2 years after MWA. The secondary outcomes included disease-free survival (DFS) and overall survival (OS) rates.

Complete ablation was defined as the postoperative contrast-enhanced MRI showing an ablative margin of at least 0.5–1.0 cm for tumors in a safe location, and a margin of at least 0.3 cm for tumors in subcapsular or perivascular locations.

### Identification of research and confounding variables

Postablation fever, including postablation peak temperature and fever duration, constitutes our research variables. Considering clinical feasibility, all temperatures were measured under the armpit. Fever was defined as an axillary temperature of ≥37°C. Fever duration was defined as the time during which the body temperature was recorded as ≥37°C, calculated using the nursing temperature record sheet. The association between fever duration and VER/ER is presented in Table S2 (Supplemental Digital Content 2, http://links.lww.com/JS9/D450). Considering the similar hazard ratios for 1 day and 2 days, as well as for 3 days and 4 days, we categorized fever duration into four groups: afebrile after ablation (0 days), fever lasting 1–2 days, fever lasting 3–4 days, and fever duration ≥5 days.

Covariates of interest included were age, sex, comorbidity, etiology, Child-Pugh classification, BCLC stage, tumor size and number, tumor differentiation, and laboratory test results. Comorbidity was assessed using the age-adjusted Charlson Comorbidity Index (aCCI)^26^ with groups of scores 0–1, 2–3, and ≥4. The BCLC stage was defined by its latest update. Tumor size and number were evaluated using the tumor burden score (TBS), calculated using the Pythagorean formula proposed by Sasaki *et al*.^27^, the formula expresses that α²=β² + γ², where α represents the TBS, β corresponds to the number of tumors, and γ denotes the maximum tumor diameter in centimeters.

If these covariates exhibited associations with VER/ER of interest and led to a change in the effect estimate exceeding 10% or had a covariate *P*-value <0.1 in the univariate model versus VER/ER, or were recommended by experts for inclusion, they were considered as confounding variables and included in the model. The associations of each confounder with VER and ER are presented in Tables S3–S4 (Supplemental Digital Content 2, http://links.lww.com/JS9/D450).

In the Kaplan–Meier curve, the confounding factors were selected based on statistical differences among groups at baseline (*P*<0.2) and recommendations from experts.

### Statistical analysis

For continuous variables, we assessed differences among the three groups using the Kruskal–Wallis test and conducted pairwise comparisons between two groups using the Wilcoxon rank-sum test. For categorical data, we applied the *χ* ^2^ test. Cumulative rates for recurrence and survival were estimated using the Kaplan–Meier method.

We initially used restricted cubic splines^28^ to visualize the nonlinear relationship between postablation temperature and VER/ER among all participants. A Log RR value of less than 0 denotes an occurrence rate for outcomes below 1. Missing values were imputed using multiple methods. Additionally, interaction analyses pertaining to postablation peak temperature were performed. We calculated hazard ratios (HRs) and 95% CIs using Cox proportional hazards models, adjusting for confounding covariates selected through stepwise regression, to assess the risk of VER/ER associated with postablation peak temperature and fever duration. Besides, protective effects were assessed across subgroups using Cox models.

For the comparison of DFS and OS among different groups, inverse probability of treatment weighting (IPTW) was used to balance the baseline characteristics. The balance in these characteristics was examined by the standardized mean difference (SMD). Adequate balance was considered achieved with an SMD lower than 0.1.

All analyses were performed and visualized using R 4.1.2 (https://www.R-project.org/), Empower (R) (www.empowerstats.com; XandY Solutions, Inc.) and GraphPad 9.0 (https://www.graphpad.com). The significance threshold was set at a two-tailed *P*-value of <0.05.

## Results

### Baseline characteristics of study participants

**Figure 1:**
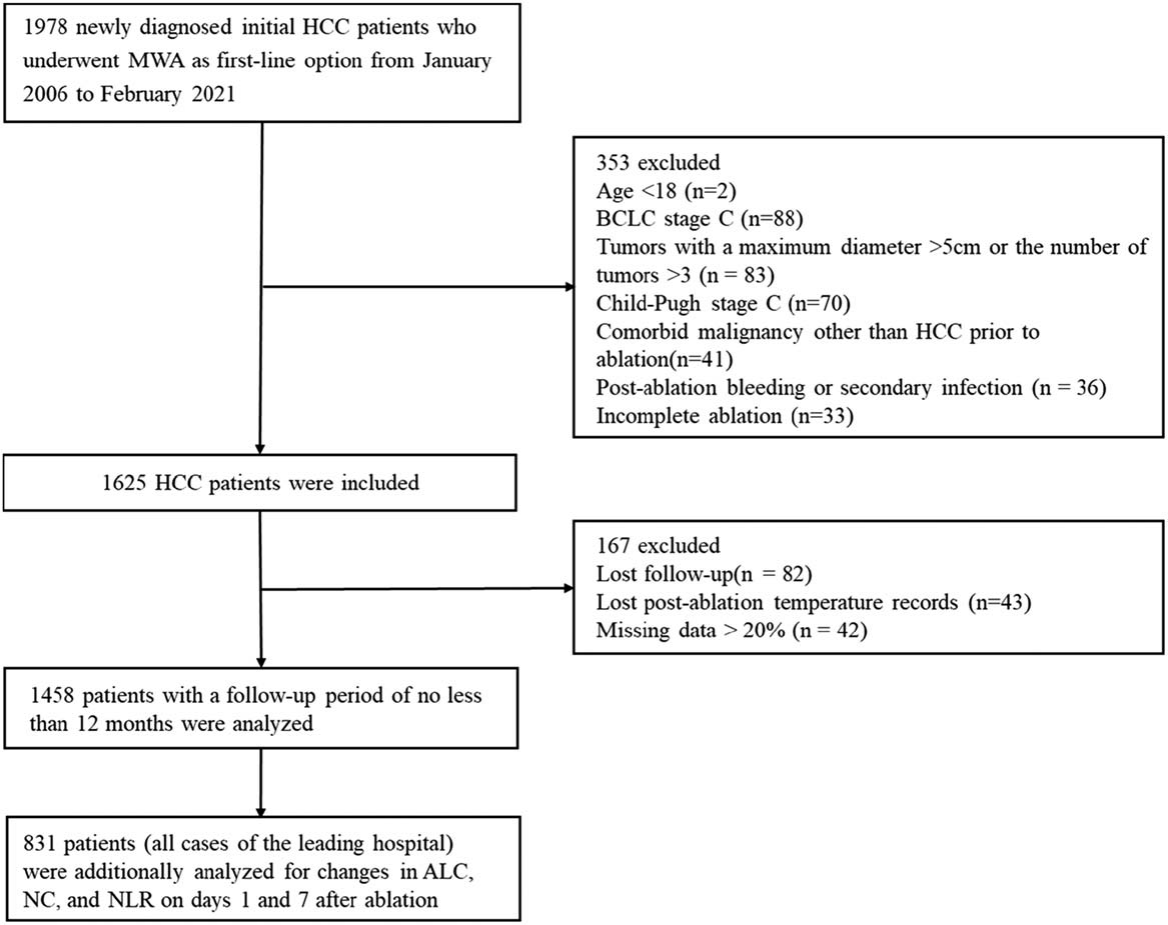
Flowchart shows patient selection. ALC, absolute lymphocyte counts; HCC, hepatocellular carcinoma; MWA, microwave ablation; NC, neutrophil counts; NLR, neutrophil-to-lymphocyte ratio.

In total, 1458 patients (mean age, 59 years±11[SD], 1146 men) with available postablation temperature data and a minimum of 1-year postablation follow-up were included in our study (Fig. 1). The demographic, clinical, and tumor characteristics, of laboratory data, and temperature record are listed in Table 1.

**Table 1:** Patient and tumor characteristics.

Characteristic	Total (*n*=1458)
Demographic and clinical data	
Age (years)*	59±11
Sex	
Male	1146 (78.6)
Female	312 (21.4)
CCI	
0–1	499 (34.2)
2–3	528 (36.2)
≥4	431 (29.6)
Etiology	
Nonviral	197 (13.5)
Viral	1261 (86.5)
Child-Pugh classification	
A	1394 (95.6)
B	64 (4.4)
BCLC stage	
0-A	1276 (87.5)
B	182 (12.5)
Tumor data	
Tumor number	
Single	1076 (73.8)
2/3	382 (26.2)
Maximum diameter (cm)*	3.2±0.9
TBS*	3.5±0.9
Differentiation	
Well	337 (23.1)
Moderate	509 (34.9)
Poor	176 (12.1)
Undetermined	436 (29.9)
Laboratory data	
AFP (ng/ml)*	10.7 (3.6–80.2)
ALB (g/l)*	38.9±4.9
TB, (mg/dl)*	14.4 (10.9–20.1)
ALT (IU/l)*	26.2(18.5–40.2)
INR*	1.1±0.2
PLT (×10^9^/l)*	125.8±62.1
WBC (×10^9^/l)*	4.9±2.0
NLR*	2.1±1.6
Postablation peak temperature (°C)	37.7±0.9
Fever duration (days)	1.0 (0–3.0)

Note. — Unless otherwise noted, data are numbers of participants, with percentages in parentheses.

* Data are mean±SD or median (Q1–Q3).

AFP, α-fetoprotein; ALB, albumin; ALT, alanine aminotransferase; BCLC, Barcelona Clinic Liver Cancer; CCI, Charlson comorbidity index; INR, international normalized ratio; NLR, neutrophil to lymphocyte ratio; PLT, platelet; TB, total bilirubin; TBS, tumor burden score; WBC, white blood cell.

**Figure 2:**
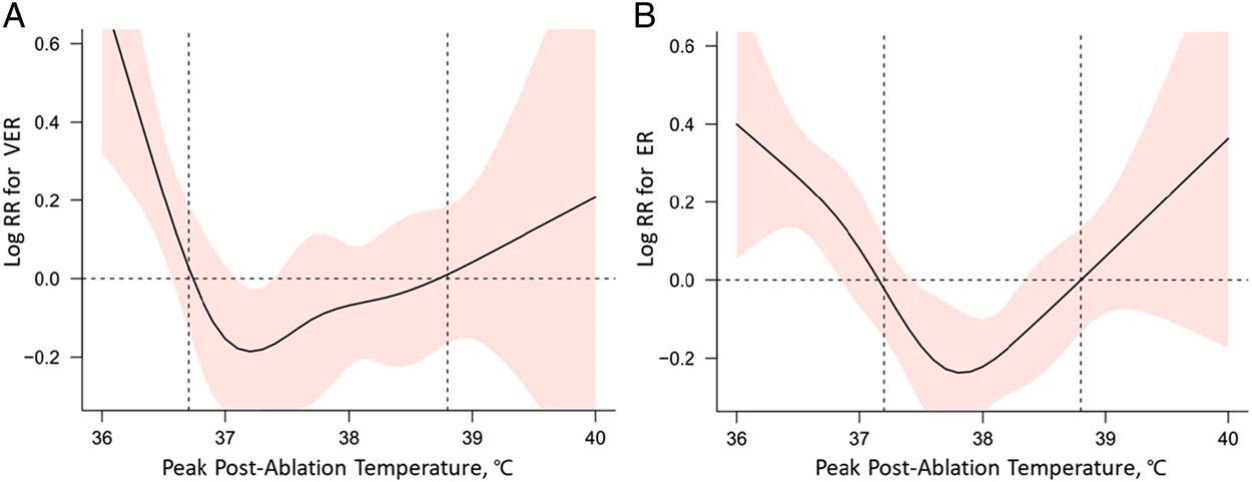
Restricted cubic splines visualized the association between postablation peak temperature and (A) very early recurrence (VER) /(B) early recurrence (ER).

The mean (SD) postablation peak temperature was 37.7°C (0.9°C), with a range of 36°C to 40°C. We employed restricted cubic splines to model and visualize the correlation between the highest temperature after ablation and both VER and ER, which revealed U-shaped correlations in both, as shown in Figure 2. After adjusting for confounding variables, the U-shaped curve still persists (Fig. S1, Supplemental Digital Content 3, http://links.lww.com/JS9/D451). We determined the temperature at which the logarithm of the risk ratio (RR) reaches 0, establishing it as the cut-off point. Subsequently, patients were categorized into three temperature ranges: 36.0–36.9°C, 37.0–38.8°C, and 38.9–40.0°C.

### The role of postablation peak temperature and fever duration in very early recurrence and early recurrence

A total of 364 individuals (25.0% of 1458) experienced very early recurrence (VER), while 580 individuals (44.9% of 1293) experienced early recurrence (ER) after ablation.

**Figure 3:**
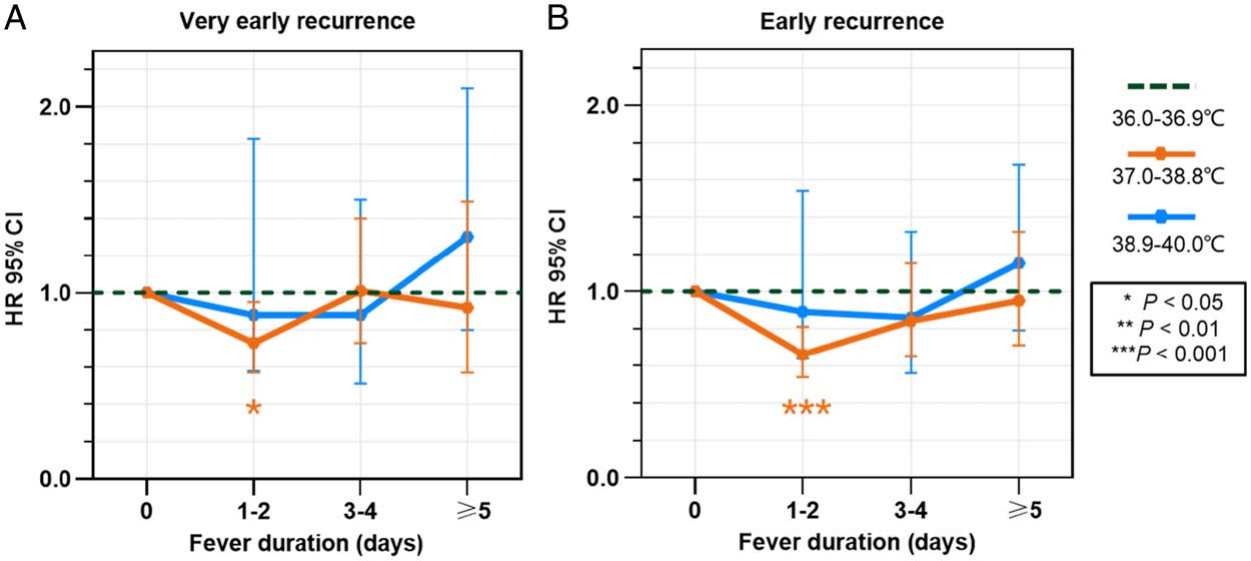
Subtypes of postablation peak temperature and fever duration and their associations with (A) very early recurrence and (B) early recurrence.

Taking into account the interaction between postablation peak temperature and fever duration’s impact on the outcomes, we categorized patients into seven groups by combining three temperature ranges and four fever durations. Our multivariate Cox regression model revealed that the combination of a temperature range of 37.0–38.8°C with a fever duration of 1–2 days showed the lowest incidence rates and emerged as an independent protective factor for both VER and ER. This resulted in a 27% reduced risk of HCC recurrence after adjusting for confounding factors (model 3, HR: 0.73, 95% CI: 0.57–0.95, *P*=0.02) in cases of VER, and a 34% reduced risk of HCC recurrence (model 3, HR: 0.66, 95% CI: 0.54–0.81, *P*<0.001) in cases of ER when compared to nonfever patients (refer to Tables 2–3 and Fig. 3).

**Table 2:** VER group.

Peak postablation temperature (°C)	36.0–36.9	37.0–38.8	38.9–40.0
All patients	399 (27.4)	909 (62.3)	150 (10.3)
VER	119 (29.8)	204 (22.4)	41 (27.3)
Model 1†	1.0	0.72 (0.58–0.90) **	0.95 (0.66–1.35)
Model 2†	1.0	0.72 (0.59–0.92) **	0.95 (0.67–1.36)
Model 3†	1.0	0.81 (0.64–1.02)	1.02 (0.70–1.46)
Fever 1–2 days			
Patients	0	652 (44.7)	24 (1.6)
VER	0	129 (19.8)	5 (20.8)
Model 1†	0	0.63 (0.49–0.80) ***	0.79 (0.49–1.74)
Model 2†	0	0.64 (0.50–0.82) ***	0.81 (0.57–1.73)
Model 3†	0	0.73 (0.57–0.95) *	0.88 (0.58–1.83)
Fever 3–4 days			
Patients	0	184 (12.6)	59 (4.0)
VER	0	55 (29.9)	16 (27.1)
Model 1†	0	0.99 (0.72–1.37)	0.88 (0.52–1.49)
Model 2†	0	1.02 (0.74–1.40)	0.83 (0.49–1.41)
Model 3†	0	1.01 (0.73–1.40)	0.88 (0.51–1.50)
Fever ≥5 days			
Patients	0	73 (5.0)	67 (4.6)
VER	0	20 (27.4)	20 (29.9)
Model 1†	0	0.94 (0.59–1.51)	1.09 (0.68–1.76)
Model 2†	0	0.97 (0.61–1.56)	1.23 (0.76–1.97)
Model 3†	0	0.92 (0.57–1.49)	1.30 (0.80–2.10)

Note. — Unless otherwise indicated, data in parentheses are percentages.

Postoperative temperature of 36–36.9°C and fever for 0 days were used as the reference for comparisons with other levels of postablation temperature and duration of fever.

The symbols *, **, and *** represent statistical significance with *P*-values less than 0.05, 0.01, and 0.001, respectively.

† The regression model provides the hazard ratio for predicting the occurrence of VER, and the data in parentheses represent the 95% CI.

BCLC stage, tumor burden score, tumor differentiation, serum AFP (<20, 20–100, >100 ng/ml), PLT, and ALB; Model 1, unadjusted; Model 2, adjusted for age and sex; Model 3, Model 2 plus an additional adjustment for the etiology.

**Table 3:** ER group.

Peak postablation temperature (°C)	36.0–36.9	37.0–38.8	38.9–40.0
All patients	323 (25.0)	827 (64.0)	143 (11.1)
ER	175 (54.2)	334 (40.4)	71 (49.7)
Model 1†	1.0	0.66 (0.55–0.79) ***	0.88 (0.67–1.16)
Model 2†	1.0	0.68 (0.56–0.82) ***	0.91 (0.69–1.20)
Model 3†	1.0	0.71 (0.59–0.85) ***	0.93 (0.70–1.24)
Fever 1–2 days			
Patients	0	587 (71.0)	24 (16.8)
ER	0	221 (37.6)	10 (41.7)
Model 1†	0	0.60 (0.49–0.73) ***	0.90 (0.67–1.56)
Model 2†	0	0.61 (0.50–0.75) ***	0.88 (0.62–1.55)
Model 3†	0	0.66 (0.54–0.81) ***	0.89 (0.64–1.54)
Fever 3–4 days			
Patients	0	173 (20.9)	57 (39.9)
ER	0	83 (48.0)	28 (49.1)
Model 1†	0	0.84 (0.65–1.09)	0.85 (0.57–1.27)
Model 2†	0	0.87 (0.67–1.12)	0.83 (0.56–1.24)
Model 3†	0	0.84 (0.65–1.10)	0.84 (0.56–1.28)
Fever ≥5 days			
Patients	0	67 (8.1)	62 (43.4)
ER	0	30 (44.8)	33 (53.2)
Model 1†	0	0.78 (0.53–1.15)	1.01 (0.69–1.46)
Model 2†	0	0.83 (0.56–1.22)	1.13 (0.78–1.64)
Model 3†	0	0.75 (0.51–1.11)	1.15 (0.79–1.68)

Note. — Unless otherwise indicated, data in parentheses are percentages.

Postoperative temperature of 36–36.9°C and fever for 0 days were used as the reference for comparisons with other levels of peak postablation temperature and fever duration.

The symbols *, **, and *** represent statistical significance with *P*-values less than 0.05, 0.01, and 0.001, respectively.

† The regression model provides the hazard ratio for predicting the occurrence of ER, and the data in parentheses represent the 95% CI.

BCLC stage, tumor burden score, tumor differentiation, serum AFP (<20, 20–100, >100 ng/ml), PLT, and NLR; Model 1, unadjusted; Model 2, adjusted for age and sex; Model 3, Model 2 plus additional adjustment for the etiology.

### Different temperature variations and patient survival

Based on the protective temperature thresholds identified in the previous section, we reclassified the different temperature variation patients into three groups: ‘afebrile’, ‘transient low-grade fever’ (TLF), included temperatures ranging from 37.0°C to 38.8°C with a fever duration of 1–2 days, and ‘prolonged or high-grade fever’ (PHF), which encompassed patients with fever lasting ≥3 days within the 37.0–38.8°C range and all patients within the 38.9–40.0°C temperature range. IPTW procedures were applied to balance baseline data among the three groups (see Fig. S2, Supplemental Digital Content 4, http://links.lww.com/JS9/D452), and both unadjusted and IPTW-adjusted variables are presented in Table 4.

**Table 4:** Patient and tumor baseline characteristics.

Characteristic	Unadjusted			*P*	IPTW-Adjusted			*P*
	Afebrile group (*n*=399)	TLF group (*n*=652)	PHF group (*n*=407)		Afebrile group (*n*=399)	TLF group (*n*=652)	PHF group (*n*=407)	
Patient characteristics								
Age (years)				0.66				0.97
18–60	198 (49.6)	341 (52.3)	213 (52.3)		51.7	51.2	50.9	
>60	201 (50.4)	311 (47.7)	194 (47.7)		48.3	48.8	49.1	
Sex				0.20				0.93
Male	326 (81.7)	503 (77.1)	317 (77.9)		77.9	78.7	77.8	
Female	73 (18.3)	149 (22.9)	90 (22.1)		22.1	21.3	22.2	
CCI				0.18				0.999
0–1	154 (38.6)	207 (31.7)	138 (33.9)		33.6	33.8	34.0	
2–3	140 (35.1)	237 (36.3)	151 (37.1)		36.7	36.4	36.0	
≥4	105 (26.3)	208 (31.9)	118 (29.0)		29.7	29.9	30.0	
Etiology				<0.001				0.98
Nonviral	77 (19.3)	82 (12.6)	38 (9.3)		13.0	13.3	13.5	
Viral	322 (80.7)	570 (87.4)	369 (90.7)		87.0	86.7	86.5	
Child-Pugh classification				0.38				0.26
A	379 (95.0)	621 (95.2)	394 (96.8)		95.1	95.2	97.1	
B	20 (5.0)	31 (4.8)	13 (3.2)		4.9	4.8	2.9	
BCLC stage				<0.001				0.98
0-A	348 (87.2)	598 (91.7)	330 (81.1)		87.8	87.6	87.4	
B	51 (12.8)	54 (8.3)	77 (18.9)		12.2	12.4	12.6	
Tumor data								
TBS*	3.6±0.9	3.4±0.9	3.7±0.9	<0.001	3.5±0.9	3.5±0.9	3.5±0.9	0.76
Differentiation				<0.001				0.999
Well	86 (21.6)	148 (22.7)	103 (25.3)		30.2	30.3	29.6	
Moderate	171 (42.9)	203 (31.1)	135 (33.2)		23.2	23.2	22.4	
Poor	61 (15.3)	64 (9.8)	51 (12.5)		34.8	34.8	35.8	
Undetermined	81 (20.3)	237 (36.3)	118 (29.0)		11.9	11.7	12.2	
Laboratory data								
AFP (ng/ml)				0.046				0.995
0–20	210 (52.6)	405 (62.1)	230 (56.5)		58.5	57.9	57.3	
20–100	85 (21.3)	114 (17.5)	81 (19.9)		19.0	19.3	18.9	
>100	104 (26.1)	133 (20.4)	96 (23.6)		22.5	22.8	23.7	
ALB (g/l)*	38.6±5.0	39.1±5.1	39.0±4.6	0.32	38.7±5.0	39.1±5.1	38.8±4.7	0.44
TB, (mg/dl)*	17.4±11.8	16.9±9.5	16.9±8.7	0.68	17.5±12.0	17.0±9.6	17.2±8.8	0.68
ALT (IU/l)*	36.2±27.1	35.4±30.2	32.1±25.5	0.08	34.7±25.1	34.8±29.4	34.9±31.5	0.99
INR	1.1±0.2	1.1±0.2	1.1±0.1	0.30	1.1±0.2	1.1±0.2	1.1±0.1	0.95
PLT(×10^9^/l)*	127.6±63.2	124.9±64.1	125.3±57.7	0.78	125.5±63.1	125.8±63.5	123.8±57.6	0.86
WBC(x10^9^/l)*	5.0±2.4	4.8±1.9	4.9±1.8	0.25	4.9±2.2	4.8±2.0	4.9±1.9	0.95
NLR*	2.2±1.8	2.0±1.6	2.0±1.4	0.07	2.1±1.5	2.1±1.6	2.1±1.8	0.95

Note. — Unless otherwise noted, data are numbers of participants, with percentages in parentheses.

* Data are mean±SD.

AFP, α-fetoprotein; ALT, alanine aminotransferase; BCLC, Barcelona Clinic Liver Cancer; CCI, Charlson comorbidity index; NLR, neutrophil to lymphocyte ratio; TBS, tumor burden score.

By December 2022 (median follow-up: 47 months, IQR: 26–79), 859 patients (58.9%) experienced tumor progression, with cumulative LTP, IDM, and EM rates of 8.2, 54.1, and 16.9%, respectively. Out of the total, 953 patients (65.4%) were alive, while 505 (34.6%) had passed away.

**Figure 4:**
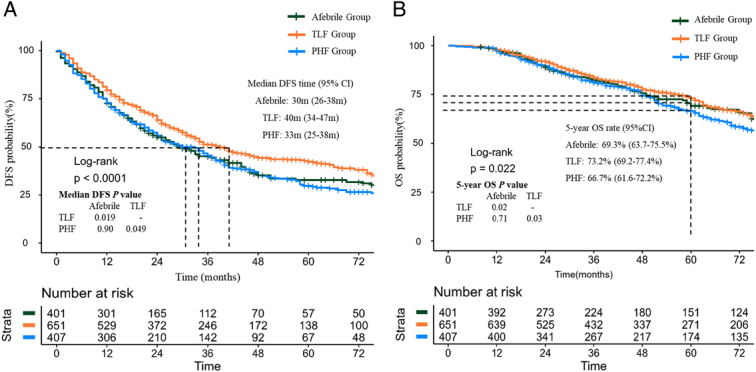
Inverse probability of treatment weighted (IPTW) adjusted cohort. (A), disease-free survival (DFS) (B), overall survival (OS).

**Figure 5:**
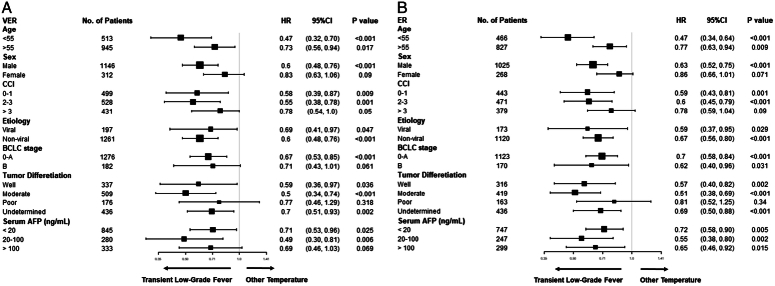
Subgroup analyses for comparing postablation TLF versus other temperature patients in (A) very early recurrence (VER) and (B) early recurrence (ER).

The Kaplan–Meier analysis revealed statistical differences among the three groups in terms of disease-free survival (DFS) and overall survival (OS) over the entire follow-up period (IPTW-adjusted overall *P*-values were <0.001 and=0.02, respectively, TLF *P*=0.01 and 0.02 vs. afebrile, and *P*<0.001 and=0.03 vs. PHF) (Fig. 5). Consistent results were obtained when analyzing the unadjusted data (see Fig. S3, Supplemental Digital Content 5, http://links.lww.com/JS9/D453). The median DFS for TLF patients was 40 months, superior to afebrile (30 months, *P*=0.019) and PHF patients (33 months, *P*=0.049), with no statistical differences observed between the afebrile and PHF patients (*P*=0.90) (Fig. 4A). The TLF group also displayed the highest 5-year OS rates of 73.2%, surpassing afebrile (69.3%, *P*=0.02) and PHF group (66.7%, *P*=0.03), with no statistical differences observed between the afebrile and PHF group (*P*=0.71) (Fig. 4B).

### Subgroup analyses

The development and prognosis of HCC are influenced by various factors. To validate the robustness of the protective effect of TLF on VER and ER, we conducted subgroup analyses based on several classic prognostic indicators, as shown in Figure 5.

In most subgroups, TLF was an independent protective factor. In the female and serum AFP >100 ng/ml subgroups, we initially observed a protective trend in the VER, although statistical significance was not reached (*P*=0.09 and 0.07). However, with longer follow-up and increased recurrence events, we found statistically significant protective effects in these subgroups for ER (*P*=0.049 and 0.02). Additionally, in the poorly differentiated HCC subgroup, TLF showed a protective trend for both VER and ER, though the results did not reach statistical significance (*P*=0.32 and 0.34).

### Dynamics of inflammatory and immune markers in ablation patients with temperature responses

In our study, we analyzed the peripheral complete blood count results of ablation patients at various time points: 1 day before, 1 day after, and at a median of 7 days postablation (IQR 5–8 days) at the leading hospital. We compared inflammatory markers, neutrophil count, and neutrophil-to-lymphocyte ratio (NLR), as well as immune marker, ALC, in three groups.

**Figure 6:**
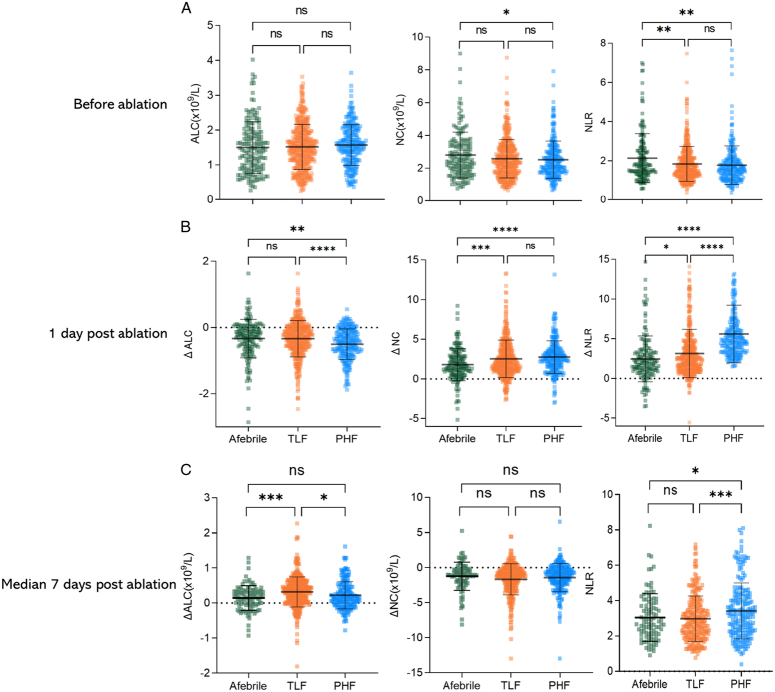
Dynamics changes of ALC, NC, and NLR in different temperature variation groups after ablation. (A), Before ablation (B), 1-day postablation (C), Median 7 days postablation.

Before the ablation, there were no significant differences in ALC among the three groups. However, the afebrile group had higher NLR compared to the TLF and PHF groups (*P*=0.001 and 0.002) (see Fig. 6A).

One day postablation, all groups showed varying degrees of ALC decline, with the PHF group experiencing the most significant drop (*P*=0.001 vs. afebrile, *P*<0.001 vs. TLF). Neutrophil counts and NLR increased to varying degrees, with the PHF group showing the most significant elevation (both *P*<0.001 vs. the afebrile group, *P*=0.22 and <0.001 vs. the TLF group) (Fig. 6B).

At a median of 7 days postablation, the TLF group showed the most significant increase in postfever ALC compared to 1-day postablation (median 3.3×10^8^/l), surpassing both afebrile and PHF patients (medians of 1.6 and 1.5×10^8^/l, *P*<0.001 and=0.013 compared to TLF, respectively). The inflammatory marker NLR remained highest in the PHF group (*P*=0.15 vs. afebrile, *P*<0.001 vs. TLF) (Fig. 6C).

## Discussion

Despite substantial advances in the management of HCC in the current era, the high recurrence rate after curative therapies remains a challenge. Our study involved 1458 BCLC stage 0-B initial HCC patients treated with percutaneous MWA at 13 tertiary hospitals over a 14-year period and discovered the relationship between postablation fever and early recurrence. We identified a U-shaped relationship between fever and early recurrence: among postablation TLF patients (with temperatures ranging from 37.0–38.8°C for 1–2 days), VER and ER were significantly lower (*P*=0.02 and <0.001, respectively), while PHF patients showed no significant differences in VER and ER compared to afebrile patients (*P*=0.96 and=0.17, respectively). In long-term survival analysis, TLF also outperformed afebrile and PHF patients in 5-year DFS and OS. Importantly, TLF was in close relationship with short-term elevated lymphocyte counts indicated a potential positive immune response contributing to improved outcomes.

Overall, the postablation fever rate in our cohort was 72.6% (1059 of 1458 patients), and the co-infection rate was only 1.4% (20 of 1059 patients), were comparable with those previously reported^18,19,29^. Limited research exists on the relationship between noninfectious postablation fever and prognosis. Only Ho *et al*.^22^ reported patients with prolonged postablation fever had a significantly higher rate of VER (72.7 vs. 37.1%, *P*=0.002). Consistently, in our study, PHF patients also showed higher VER with TLF (28.5 vs. 19.8%, *P*=0.001), and similar VER with afebrile (28.5 vs. 29.8%, *P*=0.96). In comparison, our study supplements the existing research on the relationship between TLF and prognosis. To enhance the interpretability of the prognostic analysis, we restricted our patient cohort to cases of initial HCC. This may account for the differences observed in recurrence rates and cut-off values between our study and others.

Classic prognostic factors, such as vascular infiltration, satellite nodule and microvascular invasion, are valuable for predicting prognosis, but they are typically available postsurgery and rarely obtained after ablation^5–7^. Our research confirms that postablation fever can serve as a complementary prognostic factor for ablation therapy, TLF indicating a favorable outcome. Sensitivity analyses were conducted as follows: first, by excluding patients who experienced preablation fever; second, by excluding patients with postablation bleeding or secondary infection; third, adjusting confounding variables in multiple regression analysis to verify the cut-off values of fever temperature and duration; forth, by using VER and ER two outcome variables to evaluate the consistency of the results; fifth, through subgroup analysis based on several classic prognostic factors to test the robustness of TLF’s protective effect on VER/ER; and sixth, in survival analyses using IPTW to balance baseline variables.

Fever, a major component of postablation syndrome, is believed to depend on the integrity of patients’ immune systems^25^ and considered a protective physiological response that can activate the immune system to assist the host in defense^20^. NLR, as a biomarker of systemic inflammation, has established that HCC patients with preoperative elevated NLR have a poorer prognosis after curative resection^30,31^. Elevated neutrophil counts have been linked to the suppression of cytolytic activity in immune cells, including activated T cells, NK cells, and others^32–34^. In our study, afebrile patients had the highest NLR before ablation, while PHF patients had the highest postablation NLR increase. Although the prognosis of the two groups was similar, intrinsic differences in the patients’ immune status may exist. In contrast, patients in the TLF group demonstrated the most substantial increase in postfever lymphocyte count at a median of 7 days, suggesting a robust activation of the immune system in the short-term. Previous research has reported^35^ that ablative treatments for HCC increase lymphocyte levels in peripheral blood and identify NK cells as their primary target within the lymphocyte population. Patients with a better peripheral blood NK cell response following thermal ablation also showed improved clinical outcomes, which is consistent with our study. In our study, it was observed that patients in the afebrile and PHF groups exhibited relatively weaker immune protective responses, underscoring the imperative need to enhance immune protection and concurrently implement proactive anti-tumor interventions for these individuals.

Our study has several limitations that should be acknowledged. First, our analysis relies on retrospective data, which may introduce selection bias and limit causal inference. Second, while we adjusted for confounding variables, unmeasured factors may still influence the observed associations. Third, the mechanisms underlying the observed temperature-recurrence relationships warrant further investigation.

## Conclusion

Our study highlights the importance of postablation peak temperature and fever duration in predicting HCC recurrence. A temperature range of 37.0–38.8°C with a fever duration of 1–2 days emerged as a protective factor for both VER and ER, ultimately contributing to extended OS. These findings provide valuable insights for risk stratification and management strategies in HCC patients undergoing ablation therapy. Additionally, our analysis of inflammatory and immune markers underscores the potential role of immune responses in the context of fever duration and temperature variations.

## Ethical approval

The research was conducted in compliance with the Declarations of Helsinki and Istanbul. Approval for the multi-institutional study was obtained from the Institutional Review Board at Chinese PLA General Hospital (No.020/2012).

## Consent

Due to the retrospective nature of the study, which did not involve any additional interventions, informed consent was exempt.

## Source of funding

Not applicable.

## Author contribution

Q.C.: protocol and project development, methodology, data collection, manuscript writing, and manuscript editing; C.P.: protocol and project development, methodology, and manuscript editing; Z.W., J.-M.L., Y.-Q.D., F.-Y.F., and Z.-Q.W.: protocol and project development, and methodology; X.H.: protocol and methodology; L.-J.L., X.-W.C., and R.J.: data collection and methodology; Q.M. and C.Z.: protocol; P.L., X.-L.Y., F.-Y.L., and Z.-G.C.: project development; J.Y.: protocol and project development, manuscript editing, and manuscript review. All authors contributed to the article and approved the submitted version.

## Conflicts of interest disclosure

The authors declare that the research was conducted in the absence of any commercial or financial relationships that could be construed as a potential conflict of interest.

## Research registration unique identifying number (UIN)

NCT03045952 (https://clinicaltrials.gov/).

## Guarantor

Jie Yu, MD, Department of Interventional Ultrasound, Fifth Medical Center of Chinese PLA General Hospital, 28# Fuxing Road, Beijing 100853, People’s Republic of China. Tel.: 86 10 66939530; fax: 86 10 88210006. E-mail: jiemi301@163.com.

## Data availability statement

The data that support the findings of this study are available on request from the corresponding author upon reasonable request.

## Provenance and peer review

Not commissioned, externally peer-reviewed.

## Supplementary Material
