# Supplementary material for: Relationship between postablation fever and prognosis in initial hepatocellular carcinoma: a 15-year multicenter, retrospective cohort study
Source: Int J Surg. 2024 Sep 18;111(1):962–71. doi: 10.1097/JS9.0000000000002066 (PMC11745605; doi:10.1097/JS9.0000000000002066)
Supplement: Supplementary file 2 [file js9-111-0962-s002.docx]

**Supplemental Material**

**Appendix S1**

**Summary of Operators’ Experience in the Participating Institutions**

Two operators concurrently treated a hospitalized patient, with the operator performing microwave ablation (MWA) consisting of clinical or imaging experts holding senior professional titles. Among these experts, there were 23 males and 13 females, with a median age of 45.5 years (ranging from 37 to 60). They possessed a median working experience of 24 years (ranging from 13 to 43), a median of 11.5 years (ranging from 5 to 25) of independent ablation experience, and a median annual total of 225 cases (ranging from 50 to 750) of liver tumor ablation.

**Appendix S2**

**Microwave Ablation Procedure**

The MWA system applied in this study was the KY-2000 2450 MHz microwave system (KY2000, Canyon Medical Instruments) and microwave ablation therapy equipment monitoring software. Following sedatives and local anesthesia administration, one or two 15-gauge cooled-tip antennae (11mm or 5mm front pole, 1.9mm in diameter) (Kangyou Medical, Nanjing, China) were inserted into the tumor. Microwave energy of 40–60 W was delivered for 3–10 minutes for each application. One antenna was used for tumors <2.0 cm, and two antennae were used simultaneously for tumors ≥2.0 cm. The applicators were repeatedly inserted into different sites of the tumors, if necessary, to ensure that the entire tumor could be enveloped by the estimated ablation volume assessed by intra-procedure contrast-enhanced US. The target MWA endpoint was an ablative margin of at least 0.5–1.0 cm for tumors in a safe location, complete ablation, and less than a 0.5-cm margin for tumors in subcapsular or perivascular location.

**Table S1**

**Information about Participating Institutions**

|  | Participating Institutions |
| --- | --- |
| 1 | The First Medical Center of PLA General Hospital |
| 2 | The Fifth Medical Center of PLA General Hospital |
| 3 | Cancer Hospital Chinese Academy of Medical Sciences |
| 4 | The First Bethune Hopsital of Jilin University |
| 5 | Fujian Provincial Hospital |
| 6 | The First Affiliated Hospital of Guangxi Medical University |
| 7 | The Second Affiliated Hospital of Guangxi Medical University |
| 8 | Guangxi Tumour Hospital |
| 9 | Xijing Hospital |
| 10 | Sichuan Cancer Hospital |
| 11 | The First Affiliated Hospital, College of Medicine, Zhejiang University |
| 12 | The Second Affiliated Hospital, College of Medicine, Zhejiang University |
| 13 | The Third Affiliated Hospital of Sun Yat-sen University |

**Table S2**

**Unadjusted Data for the Relationship Between Patients with Various Fever Durations and VER/ER**

|  | VER Group | | ER Group | |
| --- | --- | --- | --- | --- |
|  | HR (95%CI) | *P* value |  | *P* value |
| 37.0-38.8℃ Fever duration(days) |  |  |  |  |
| 1 | 0.62(0.47,0.82) | <.001 | 0.62(0.50,0.77) | <.001 |
| 2 | 0.63(0.44,0.90) | .01 | 0.56(0.42,0.74) | <.001 |
| 3 | 1.04(0.73,1.46) | .84 | 0.80(0.60,1.07) | .14 |
| 4 | 0.86(0.46,1.60) | .63 | 0.98(0.63,1.53) | .93 |
| ≥5 | 0.94(0.58,1.50) | .79 | 0.78(0.53,1.15) | .21 |
| 38.9-40.0℃ Fever duration(days) |  |  |  |  |
| 1 | 1.14(0.36,3.57) | .83 | 0.72(0.27,1.95) | .52 |
| 2 | 0.46(0.11,1.84) | .27 | 0.66(0.29,1.48) | .31 |
| 3 | 1.13(0.57,2.22) | .73 | 0.85(0.47,1.52) | .59 |
| 4 | 0.69(0.32,1.48) | .35 | 0.85(0.51,1.42) | .54 |
| ≥5 | 1.09(0.68,1.74) | .73 | 1.01(0.70,1.46) | .96 |

**Table S3**

**Univariate Analysis of Covariates for the VER and ER Groups**

| Covariates | VER Group | *P* value | ER Group | *P* value |
| --- | --- | --- | --- | --- |
| CCI |  |  |  |  |
| 0-1 | *Reference* | *Reference* | *Reference* | *Reference* |
| 2-3 | 1.09(0.84,1.42) | 0.52 | 1.15(0.93,1.42) | .19 |
| ≥ 4 | 1.13(0.85,1.51) | .39 | 1.19(0.94,1.49) | .14 |
| Etiology |  |  |  |  |
| Viral | *Reference* | *Reference* | *Reference* | *Reference* |
| Non-viral | 0.63(0.48,0.84) | .002 | 0.78(0.62,1.00) | .048 |
| Child-Pugh classification |  |  |  |  |
| A | *Reference* | *Reference* | *Reference* | *Reference* |
| B | 1.23(0.79,1.91) | .36 | 1.06(0.73,1.54) | .76 |
| BCLC stage |  |  |  |  |
| 0-A | *Reference* | *Reference* | *Reference* | *Reference* |
| B | 1.36(1.06,1.75) | .02 | 1.17(0.95,1.43) | .15 |
| TBS | 1.18(1.06,1.33) | .004 | 1.11(1.02,1.22) | .02 |
| Differentiation |  |  |  |  |
| Undetermined | *Reference* | *Reference* | *Reference* | *Reference* |
| Well | 0.86(0.63,1.17) | .34 | 0.92(0.73,1.16) | .47 |
| Moderate | 1.17(0.90,1.53) | .24 | 1.02(0.83,1.26) | .83 |
| Poor | 1.44(1.06,1.97) | .02 | 1.12(0.86,1.44) | .41 |
| Serum AFP (ng/mL) |  |  |  |  |
| <20 | *Reference* | *Reference* | *Reference* | *Reference* |
| 20-100 | 1.14(0.87,1.49) | .35 | 1.11(0.90,1.36) | .32 |
| >100 | 1.52(1.20,1.94) | <.001 | 1.10(0.90,1.35) | .35 |
| ALB (g/L) | 0.97(0.95,0.99) | .01 | 0.99(0.98,1.01) | .32 |
| TB, (mg/dL) | 1.00(0.99,1.01) | .50 | 1.00(1.00,1.01) | .36 |
| ALT (IU/L) | 1.00(0.99,1.01) | .34 | 1.00(1.00,1.00) | .97 |
| INR | 1.16(0.62,2.17) | .64 | 0.92(0.55,1.55) | .76 |
| PLT (x10^9^/L) | 1.00(1.00,1.00) | .78 | 1.00(1.00,1.00) | .61 |
| WBC (x10^9^/L) | 0.99(0.95,1.05) | .82 | 1.02(0.98,1.06) | .43 |
| Lymphocyte(x10^9^/L) | 1.04(0.89,1.20) | .64 | 0.97(0.86,1.09) | .63 |
| NLR | 1.01(0.95,1.07) | .80 | 1.04(0.99,1.08) | .10 |

Note. —Univariate model of covariates also adjusted for fixed covariates: age and gender. Values are Hazard ratio (95% Confidence Interval) from univariate regression models based on Cox model and reflect differences in VER and ER per unit change of each covariate and for different categories of each covariate as compared to the reference group

**Table S4**

**Introducing covariates in the baseline model and removing covariates in the full model to observe the change in the regression coefficient of Post-ablation Peak Temperature (℃)**

|  | VER Group | | ER Group | |
| --- | --- | --- | --- | --- |
| Covariates | Basic model | Full model | Basic model | Full model |
| +/- term | X | X | X | X |
| IRC | -0.1105 | -0.0727 | -0.0506 | -0.0401 |
| CCI | -0.1110 | -0.0740 | -0.0510 | -0.0416 |
| Etiology | -0.0947 * | -0.0960 * | -0.0431 * | -0.0507 * |
| Child-Pugh classification | -0.1094 | -0.0733 | -0.0503 | -0.0402 |
| BCLC stage | -0.1213 | -0.0708 | -0.0560 * | -0.0392 |
| TBS | -0.1247 * | -0.0682 | -0.0601 * | -0.0357 * |
| Differentiation | -0.0962 * | -0.0891 * | -0.0460 | -0.0445 * |
| AFP, ng/mL | -0.0967 * | -0.0849 * | -0.0485 | -0.0420 |
| ALB (g/L) | -0.1118 | -0.0708 | -0.0512 | -0.0395 |
| TB, (mg/dL) | -0.1100 | -0.0735 | -0.0501 | -0.0403 |
| ALT (IU/L) | -0.1061 | -0.0760 | -0.0506 | -0.0397 |
| INR | -0.1105 | -0.0729 | -0.0505 | -0.0402 |
| PLT (x10^9^/L) | -0.1107 | -0.0728 | -0.0508 | -0.0399 |
| WBC (x10^9^/L) | -0.1108 | -0.0740 | -0.0499 | -0.0400 |
| Lymphocyte(x10^9^/L) | -0.1113 | -0.0737 | -0.0500 | -0.0401 |
| NLR | -0.1100 | -0.0773 | -0.0441 * | -0.0425 |

Note. —Univariate model of covariates also adjusted for fixed covariates: age and gender

+/- term：add the covariate X to basic model or remove it from full model. X= post-ablation peak temperature (℃) IRC= initial regression coefficient

* The impact of the regression coefficient on X >10%
