# Supplementary material for: Relationship between postablation fever and prognosis in initial hepatocellular carcinoma: a 15-year multicenter, retrospective cohort study
Source: Int J Surg. 2024 Sep 18;111(1):962–71. doi: 10.1097/JS9.0000000000002066 (PMC11745605; doi:10.1097/JS9.0000000000002066)
Supplement: Supplementary file 4 [file js9-111-0962-s004.pptx]

## Slide 1
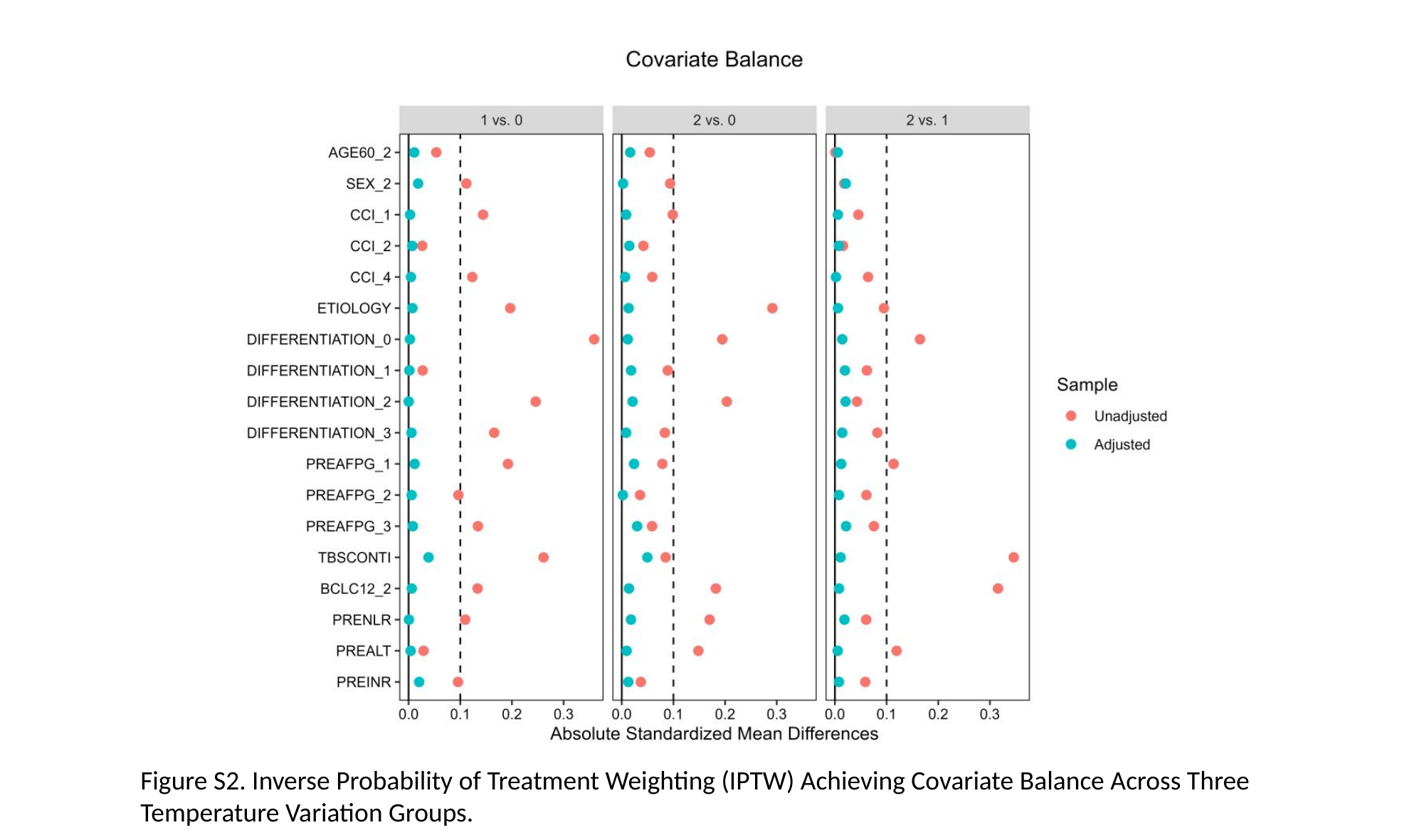

Figure S2. Inverse Probability of Treatment Weighting (IPTW) Achieving Covariate Balance Across Three
Temperature Variation Groups.
