# Supplementary figures and images for: Relationship between postablation fever and prognosis in initial hepatocellular carcinoma: a 15-year multicenter, retrospective cohort study
Source: Int J Surg. 2024 Sep 18;111(1):962–71. doi: 10.1097/JS9.0000000000002066 (PMC11745605; doi:10.1097/JS9.0000000000002066)

## Slide 1
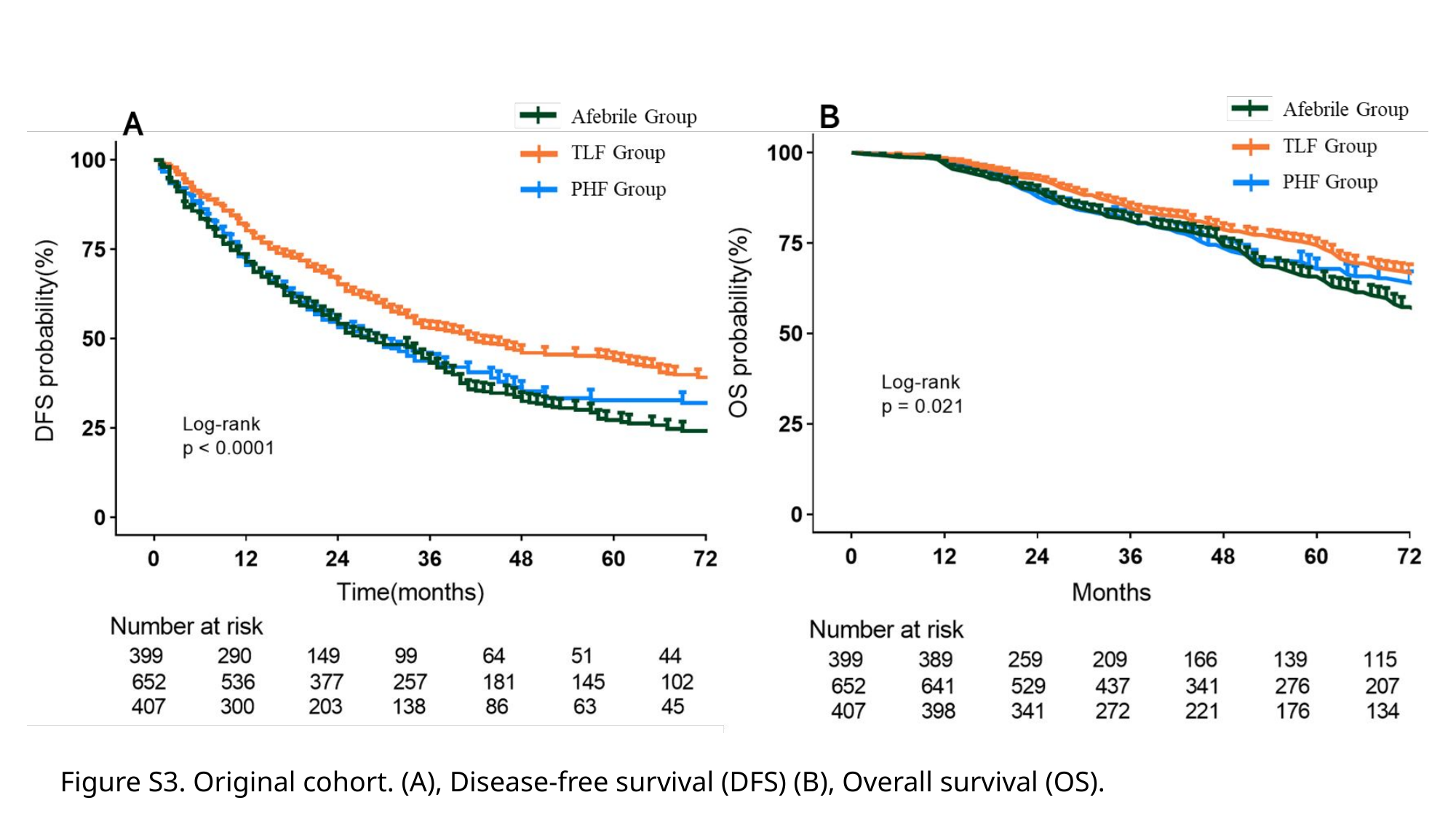

Figure S3. Original cohort. (A), Disease-free survival (DFS) (B), Overall survival (OS).

Supplement: Supplementary file 5 [file js9-111-0962-s005.pptx]
